# Moving beyond the GM Debate

**DOI:** 10.1371/journal.pbio.1001887

**Published:** 2014-06-10

**Authors:** Ottoline Leyser

**Affiliations:** Sainsbury Laboratory, University of Cambridge, Cambridge, United Kingdom; The University of North Carolina at Chapel Hill, United States of America

## Abstract

Once again, there are calls to reopen the debate on genetically modified (GM) crops. I find these calls frustrating and unnecessarily decisive. In my opinion the GM debate, on both sides, continues to hamper the urgent need to address the diverse and pressing challenges of global food security and environmental sustainability. The destructive power of the debate comes from its conflation of unrelated issues, coupled with deeply rooted misconceptions of the nature of agriculture.

## Abstract 2

A secure, environmentally sustainable food supply is agreed to be a vital priority. In this Perspective, Ottoline Leyser explains why she believes that progress toward this important goal is side-tracked by unproductive debates about the role of genetic modification.

This article is part of the *PLOS Biology* Collection “The Promise of Plant Translational Research.”

For many people, genetic modification (GM) has become the poster child for everything they consider bad about modern agriculture. It represents the domination of the food supply chain by profit-driven multinational companies. It represents the systematic replacement of important ecosystems with huge high-intensity farms growing monocultures of commodity crops. It represents humankind's evil manipulation of Nature for personal gain and greed, at the expense of the planet and of future generations. These are important concerns. It is reasonable to be disturbed by some of the current trends in agricultural practices, with fears fuelled by past errors, such as the previous emergence in the UK of bovine spongiform encephalopathy (BSE). However, none of these issues has anything to do with GM as a technique for improving or introducing plant traits. A complete ban on the use of GM in crop development would have no impact on any of them. For as long as we imagine that GM itself is the cause of these problems, they are free to escalate unchecked.

A defining question of the 21^st^ century is: How can we achieve a reliable, sustainable, equitable supply of nutritious food for a growing and increasingly urbanized world population in the face of climate change? This is a complex question with agricultural productivity constituting only a small part of it, and in turn, GM only a small part of that. It is essential that we move forward to address this question without being continuously sidetracked by the GM debate. How can this be achieved?

First, it is necessary to move on from the well-worn logical fallacy that anything natural is good, and anything unnatural is bad. The application of this fallacy to agriculture is an excellent illustration of why it is so flawed. Plants evolved by natural selection, driven by the survival of the fittest. As a result, naturally, they are defended to the hilt from herbivores of all kinds, including humans. We know this. No one sends their children into the woods saying “Eat anything you find. It's all natural, so it must be good for you.” The seeds of plants are particularly well protected, because they are, of course, the plant's children, their ticket to posterity. Seed is therefore usually tough, indigestible, minimally resourced, and often laced with toxins. Yet plant seeds are now our major source of calories. The cereal crops we eat bear little resemblance to their naturally selected ancestors, and the environments in which we grow them are equally highly manipulated and engineered by us. We have, over the last 10,000 years, bred out of our main food plants all kinds of survival strategies that natural selection put in. This has drastically reduced their competitiveness in nature, but equally dramatically increased their utility in feeding us. Agriculture is the invention of humans. It is the deliberate manipulation of plants (and animals) and the environment in which they grow to provide food for us. The imperative is not that we should stop interfering with nature, but that we should interfere in the best way possible to provide a reliable, sustainable, equitable supply of nutritious food. To do this we need to understand how nature works. That's what science is all about.

This is easy to say, but concepts of the inherent goodness of Nature, and the inherent dangers of human interventions through science, are deeply ingrained in the way many people think, particularly in the context of food. This is an understandable response to concerns over the industrialization and unsustainable intensification of agriculture described above. You only have to walk down the aisles of a supermarket to see that “all natural” and “nothing artificial” sells things. These words sell products because they are so strongly culturally associated with environmental sustainability and well-being, exploiting people's interest in protecting the environment and their health. However, many of the products people think of as natural, such as cereal crops, are profoundly unnatural and wouldn't exist without human intervention; and many things people think of as artificial, such as “chemicals,” can be made with no human involvement at all. Similarly, many “natural” things are extremely bad news, such as aflatoxins, and many “artificial” things are widely accepted to be an extremely good idea, such as cereal crops (again). The only way to determine whether something is environmentally sustainable or healthy is to do the science and find out. Guessing based on cultural norms, amplified by aggressive marketing strategies is understandable, but will not deliver the desired outcome: a sustainable supply of healthy food. People need to be empowered to make decisions in a different way. For example, in the UK there is now a well-established health wheel traffic light system on foods in supermarkets, indicating through a simple graphic the sugar, fat, salt, and calorific content of the product. Perhaps there could be a similar sustainability wheel, building on such initiatives as Leaf (for Linking Environment and Farming – a UK organization that promotes sustainable food and farming and that identifies food produced to high environmental standards to consumers with a Leaf logo) [1]. This could be combined with the stricter application of advertising standards, preventing the fostering of misleading claims about what counts as “natural” and of misleading implications about the associated health and environmental benefits.

Second, we need to get past the idea that GM, as a technique for crop genetic improvement, is specifically and generically different from other approaches, including conventional selective breeding. GM involves introducing a gene directly into the genome of an organism. The introduced gene can be one found in other members of that species or it could be from a different species. The most distinctive generic thing about a GM crop, in comparison to one produced by conventional selective breeding, would therefore appear to be the insertion of a piece of DNA into its genome, a process that is certainly not unique to GM crops. Even the movement of genes between species is not GM-specific, and indeed GM crops need not be modified with genes from a different species. Many viruses can insert their genomes into that of their host as a normal part of their life cycle. These viral sequences, and many related genetic elements, such as retroposons, accumulate over evolutionary time and can continue to move about the genomes of their hosts, creating new DNA insertion sites. Thus, every conventionally bred rice crispy or cornflake you had for breakfast probably differs from every other one by the insertion of a piece of DNA at an unknown site in its genome.

There is really nothing generic to be said about GM as a plant breeding technique. Almost all the media reports purporting to be about the effects of GM are in fact about effects of the specific trait that has been introduced into the GM crop. Currently, there are only two widely deployed GM traits: herbicide tolerance and insect resistance. Concerns purporting to be about GM are almost all about one or other of these traits. For example, a large, farm-scale evaluation of the environmental impacts of three herbicide-tolerant GM crops conducted in the UK between 1999 and 2006 [2] was widely reported as demonstrating that GM is bad for wildlife. What in fact it showed was that effective weed control is bad for wildlife. Weeds are required to support biodiversity in agricultural environments, and are currently under threat from winter planting regimes, non-GM herbicide-tolerant crops, and a range of increasingly sophisticated weed control strategies. Banning GM crops will not address this problem. It is wrong to imply that growing GM crops, rather than effective weed control, is the cause of negative effects on biodiversity. It is not because a crop is GM that weeds are reduced; many GM crops have no impact on weeds at all. It is because a crop, GM or otherwise, is herbicide tolerant and sprayed with weed killers that reduce weed populations. The claim that biodiversity is reduced because a GM crop was grown detracts attention from the real issue, namely how to balance the positive effects of weeds in supporting biodiversity with their negative impacts on agricultural productivity.

This confusion between the effects of a new trait and the method by which it has been introduced is enshrined in the way new crops are licensed for commercial release in many countries. In the European Union (EU), a new herbicide-tolerant GM crop, produced by introducing a single gene conferring herbicide tolerance, must go through a lengthy procedure of testing aimed at assessing its potential health and environmental impacts [3]. Such an assessment would include concerns about impacts on wildlife, as described above, and about the generation of so-called super weeds by out-crossing of the GM crop to wild relatives or caused by the over-use of herbicides. Meanwhile, a herbicide-tolerant crop produced by mutation of a single endogenous gene has no such testing, and the breeders need only to demonstrate that it is stable and significantly different from already registered crops. All the environmental concerns associated with GM herbicide tolerance are equally applicable to non-GM herbicide tolerance. There are also considerable agronomic and environmental benefits that could accrue from herbicide tolerant crops, such as reduced soil erosion through reduced need for ploughing [4]. These need to be weighed against the risks and an appropriate decision reached. This decision is about weed control, not about GM. In my opinion, there is therefore no justification for considering GM vs non-GM herbicide tolerant crops differently. Their assessment, from a regulatory viewpoint and in terms of their environmental impact, should be based on the distinctive trait they carry.

The GM-specific regulatory system currently in place creates huge financial barriers for GM crop introduction, which ironically is one of the main reasons why almost the only applications in the field today are driven by big business. These days, the cost of developing a GM crop is relatively affordable. Meanwhile, non-GM crops, sometimes with new traits, are released with relatively little scrutiny of their impacts on the environment or on food safety. This is increasingly an issue as we continue to develop new and ever more sophisticated ways to introduce desirable traits into crops, for example by genomic assisted breeding or by genome editing [5]. These new tools provide exciting and much needed opportunities for crop genetic improvement, but in my view they also demand a more sensible licensing system that assesses all new crops based on the traits they carry rather than on the method by which they were introduced [5],[6].

The current system does little to protect the environment or the food chain and is ill-equipped to cope with the new approaches to plant breeding now coming on line. A trait-based system, bringing a proportional level of scrutiny to all crops that carry a new trait, could provide the checks and balances that should go hand in hand with innovation. We definitely need crop genetic improvement [4], using whatever method is best, and it is precisely because we do that we also need an evidence-based and proportional system for assessing new crops for environmental and health impacts.

A related issue, which will be similarly challenged by new genetic improvement techniques, is that of patent protection for crops. While conventionally bred crops can be protected by various means, such “variety” protection systems include exemptions for farmers that permit them to save seed for next year's planting and for breeders to include the variety in breeding programmes. In contrast, GM crops can be protected by so-called utility patents, which can protect the use of a specific gene to confer a trait. These patents are much more restrictive and prohibit both seed saving by farmers and exemptions for plant breeders. The harmonisation of the crop variety licensing system to focus on novel traits, however introduced, could reasonably be widened to include an examination of the patent protection system for such traits. If the licensing system were to become less expensive, the argument for restrictive utility patents on such traits is reduced.

We now have a wealth of opportunities for crop genetic improvement, with an impressive arsenal of tools and techniques available. To deploy these effectively, we need to move well beyond the GM debate to a much wider debate about food production. What methods of farming provide reliable and high yields in a sustainable way? What is the role of multinational companies in delivering food security? What political and societal changes are needed to drive more equitable food distribution? How can waste be reduced? These are big complex questions with big complex answers and no simple dogmatic solution. No single farming method or crop improvement technique is a panacea, nor is it the cause of the problem. Such complex problems with correspondingly complex and multifaceted solutions are difficult. They don't make rousing campaign slogans or eye-catching tabloid headlines, but we have got to find a way to address them, in all their complexity.

The most frustrating thing about this situation is that almost everyone wants the same outcome: a reliable, sustainable, equitable supply of nutritious food. For issues this big, there will of course be differences of opinion about how to move forward, what to prioritise, and how to decide. These are important areas for debate. GM, as a technique, is not.
